# Elemental biomapping of human tissues suggests toxic metals such as mercury play a role in the pathogenesis of cancer

**DOI:** 10.3389/fonc.2024.1420451

**Published:** 2024-06-21

**Authors:** Roger Pamphlett, David P. Bishop

**Affiliations:** ^1^ Department of Neuropathology, Royal Prince Alfred Hospital, Sydney, NSW, Australia; ^2^ Hyphenated Mass Spectrometry Laboratory, School of Mathematical and Physical Sciences, University of Technology Sydney, Sydney, NSW, Australia

**Keywords:** cancer, toxic metals, mercury, neoplasia, carcinogenesis, pathogenesis, human tissue, elemental biomapping

## Abstract

Toxic metals such as mercury, lead, and cadmium have multiple carcinogenic capacities, including the ability to damage DNA and incite inflammation. Environmental toxic metals have long been suspected to play a role in the pathogenesis of cancer, but convincing evidence from epidemiological studies that toxic metals are risk factors for common neoplasms has been difficult to gain. Another approach is to map the location of potentially toxic elements in normal human cells where common cancers originate, as well as in the cancers themselves. In this Perspective, studies are summarized that have used elemental biomapping to detect toxic metals such as mercury in human cells. Two elemental biomapping techniques, autometallography and laser ablation-inductively coupled-mass spectrometry imaging, have shown that multiple toxic metals exist in normal human cells that are particularly prone to developing cancer, and are also seen in neoplastic cells of breast and pancreatic tumors. Biomapping studies of animals exposed to toxic metals show that these animals take up toxic metals in the same cells as humans. The finding of toxic metals such as mercury in human cells prone to cancer could explain the increasing global incidence of many cancers since toxic metals continue to accumulate in the environment. The role of toxic metals in cancer remains to be confirmed experimentally, but to decrease cancer risk a precautionary approach would be to reduce emissions of mercury and other toxic metals into the environment from industrial and mining activities and from the burning of fossil fuels.

## 1 Introduction

Most cancers appear to result from interactions between genetic variations and injurious environmental agents (1). Advances have been made in identifying germline and somatic gene variants that increase cancer risk, but finding convincing cancer-promoting environmental toxic agents (toxicants) for most cancers has proven difficult. Reasons for this include: (**1**) the increasing number of potential environmental agents that could play a role in cancer pathogenesis, with over 350,000 chemicals and mixtures of chemicals registered (2); (**2**) exposure to toxic agents may have been years before the cancer developed, during which time the toxicant was removed from the tissue (a “hit and run” scenario); (**3**) multiple synergistically-acting toxic agents may need to be involved before cancer develops (3, 4), i.e., a “poly-environmental” combination of risk factors; (**4**) most people are unaware of which toxic agents they have been exposed to. All of these make studies looking for toxic metals as risk factors for cancer challenging to undertake and interpret (3, 5, 6).

Some groups have looked for toxicants within tumor samples, usually using bulk chemistry methods (7–12). The difficulty here is that tumors are often supplied by new blood vessels that are permeable to circulating toxicants, which would normally only have limited access to the original tissue. The finding of toxicants in tumor tissue may therefore be a secondary phenomenon not related to cancer initiation. Animal experiments have given insights as to how exposure to some toxicants could give rise to cancer (13, 14), but the number of toxicants tested has been small, and young genetically-identical animals are often employed. It is difficult to design animal studies to examine how exposure to multiple toxicants over many years, in a genetically variable population, could result in cancers affecting humans.

**Figure 1:**
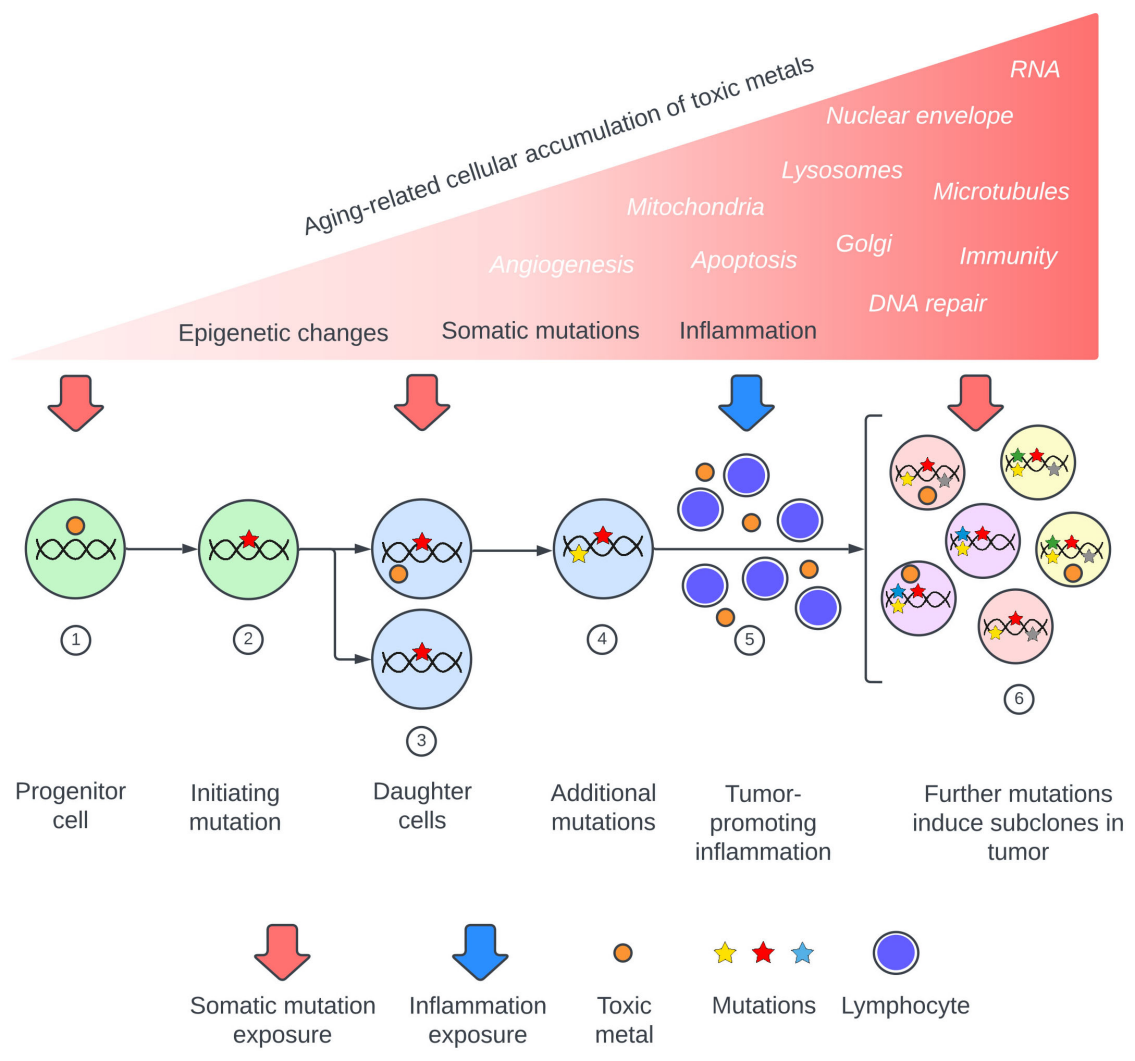
Proposed pathway of toxic metal exposure leading to cancer. *Upper section*: Toxic metal exposure arises from repeated episodes or from a constant source, with the cellular burden of toxic metals increasing during aging. Major consequences are somatic mutations, inflammation, and epigenetic changes, while toxic metal-induced alterations to intracellular processes and organelles (in white italics) can also promote carcinogenesis. *Lower section*: Examples of toxic metal exposures resulting in two of these mechanisms, somatic mutations and inflammation. (**1**) Toxic metals enter a progenitor cell and produce (**2**) a cancer-initiating mutation. (**3**) Daughter cells carrying the initiating mutation take up further toxic metals which produce driver mutations (**4**). (**5**) Circulating toxic metals initiate tumor-promoting inflammation. (**6**) Toxic metals within tumor cells produce subclone mutations.

Metal toxicants suspected to be involved in cancer pathogenesis include lead, cadmium, mercury, arsenic, and chromium (3–6, 13, 15–19). These metals are found globally in air, water, and soil (20), and exhibit many of the complex mechanisms that underlie cancers. These mechanisms include somatic mutation-inducing DNA damage (13, 17, 18, 21–24), impaired DNA repair (13, 24–26), inflammation and oxidative stress (13, 17, 24, 27, 28), epigenetic changes (4, 24, 29–32), changes to apoptosis with increased cell survival and proliferation (14, 17, 33, 34), and damage to cellular organelles and membranes such as mitochondria (35, 36), the Golgi apparatus (37, 38), lysosomes (39, 40), and nuclear envelopes (37, 41). Other toxic metal cancer-promoting mechanisms include alterations to microtubules (13, 42), increased angiogenesis (43, 44), damaged RNA (45, 46), and immune changes (47, 48). Recently published reviews of the carcinogenic potentials of toxic metals emphasize that multiple injurious modes of these metals can act together, and that synergistic actions come into play when several toxic metals are present (**Supplementary Table 1**). A model of toxic metal-promoted carcinogenesis is proposed, illustrating the extensive range of mechanisms that could be involved (**Figure 1**). The sequence in which these events occur could be important. For example, it has been proposed that early epigenetic changes in pre-cancerous lesions that allow for cell proliferation would favor subsequent genetic mutations in these cells (32, 49).

Many previous studies have looked at the possible roles of toxic metals in cancer; a recent review found 820 studies on heavy metals and cancer risk published between 2000 and 2022 alone (6). What remains lacking to support the toxic metal hypothesis for cancer is evidence that metal toxicants are present early in the cells in which neoplasms originate. In this Perspective, an overview of selected elemental bioimaging studies of human tissues is presented, concentrating on normal tissues (to look for underlying predispositions to cancer initiation) and including some neoplasms (to look for factors promoting tumor progression). Mercury is mainly used throughout the Perspective as an example of a toxicant that could play a role in carcinogenesis, since human exposure to mercury is common from both inhaled mercury vapor and ingested methylmercury (especially from eating large predatory fish) (50), the tissue distribution of mercury in experimentally-exposed animals has been well studied (51–56), both mercury vapor and methylmercury are metabolized in the body to toxic divalent mercury cations that accumulate in cells (57), mercury has many of the toxic effects suspected to underlie carcinogenesis (13, 18, 21), and mercury is the major toxic metal detected by autometallography, the technique that is used for elemental mapping of large numbers of tissue samples (58–60). Therefore, in this Perspective we will focus on studies that have looked at the cellular distribution of mercury (as a typical toxic metal) in human cells to see if these give clues to cancer pathogenesis.

## 2 Elemental biomapping of human tissues

### 2.1 Elemental biomapping techniques

Two techniques that can be used to examine the distribution of toxic elements in human cells are autometallography (AMG) and laser ablation-inductively coupled-mass spectrometry imaging (LA-ICP-MSI). (**1**) Autometallography is a physical development amplification technique (based on that first used in photography) that enables inorganic mercury, silver, or bismuth bound to selenides or sulfides in tissues to convert added silver ions (from silver nitrate or lactate) into black metallic silver, which then visibly coats even a few atoms of these metals (58–63) (referred to here as ^AMG^TM). Autometallography can be combined with immunohistochemistry (64–67) or electron microscopy (58, 61) to detect ^AMG^TM in specific cells. (**2**) LA-ICP-MSI is a multi-elemental imaging technique that uses a laser to sample histological sections, with the ablation plume swept into an ICP-MS (68). When analyzed alongside matrix-matched calibration standards, quantitative images are reconstructed from the data. LA-ICP-MSI is a powerful technique for detecting the roles metals play in cancer (69), however is not as sensitive as AMG, the detection limit being 0.05–0.81 μg per g (70). Other elemental imaging techniques are not covered in this review due to their specialized requirements. For example, synchrotron X-ray fluorescence microscopy measures concentrations of elements within cells but is limited to very small areas of frozen tissue (71). NanoSIMS is a sensitive high resolution imaging technique, but the ultra-thin samples need a high vacuum and cannot be applied to stored histological sections (72).

### 2.2 Human cells containing metal toxicants

Studies of autopsy-sampled tissues of 170 people with a variety of clinicopathological conditions [one exposed to mercury (73)], as well as surgical samples of normal tissue adjacent to neoplastic tissue, show that toxic metals are commonly present in selective groups of human cells (64–67, 74–79). Human cells that contained toxic metals were seen most often in the kidney, pancreas, thyroid, nervous system, anterior pituitary, breast, ovary, adrenal gland, liver, retina, and in endothelial cells in many organs.

#### 2.2.1 Kidney

**Figure 2:**
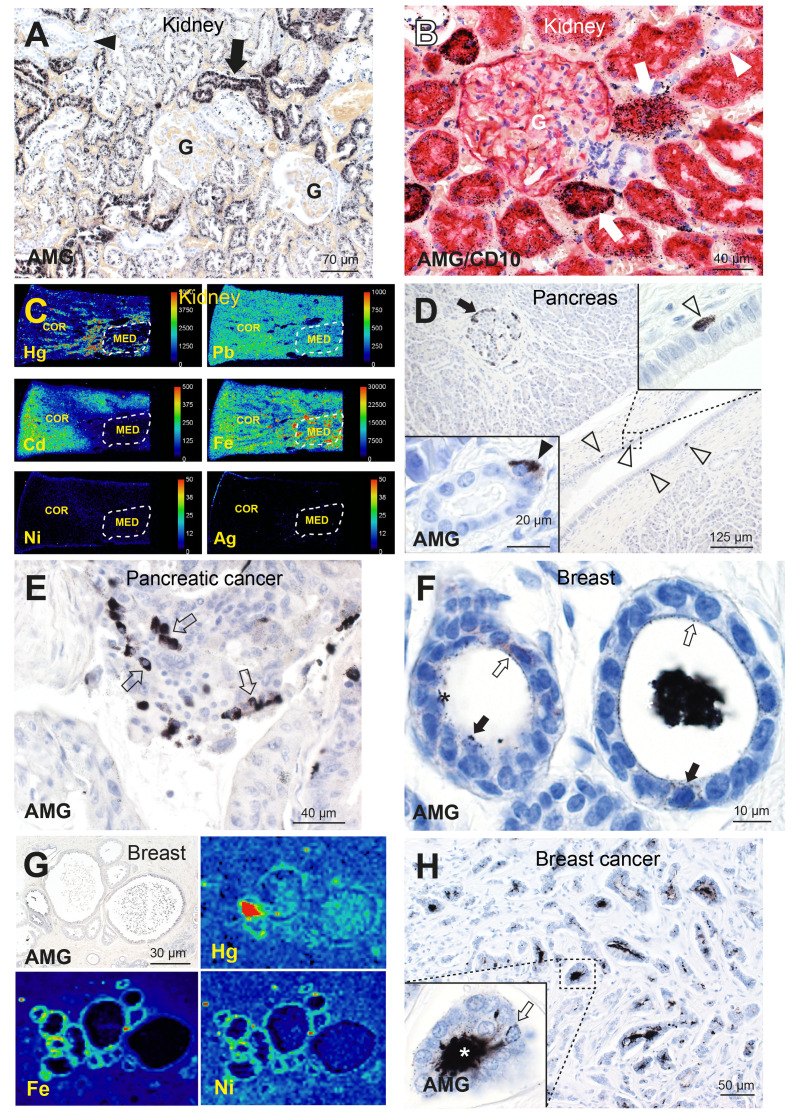
Toxic metals in the human kidney, pancreas, and breast. **(A)** A normal kidney has abundant ^AMG^TM in proximal tubule cells (arrow). No mercury is seen in glomeruli (G) or in distal tubules (arrowhead). AMG/hematoxylin (66). **(B)** AMG with CD10 immunostaining shows red proximal tubule cells containing black mercury grains (arrows). No mercury is seen in CD10-negative distal tubules (arrowhead), or in a glomerulus (G) whose cells stain lightly with CD10. AMG/CD10/hematoxylin (66). **(C)** LA-ICP-MSI shows mercury, lead, cadmium and iron in kidney cortex or medulla, but not nickel or silver (66). Scale = counts per second (proportional to abundance). CO: cortex, ME: medulla (within dashed outlines). **(D)** A pancreas with ^AMG^TM in peripheral (arrow) and internal islet cells. Scattered periductal cells (open arrowheads), one enlarged in the upper right inset, contain ^AMG^TM. *Lower left inset*: an acinar cell contains ^AMG^TM (closed arrowhead). AMG/hematoxylin (65). **(E)** Individual and groups of pancreatic carcinoma cells contain ^AMG^TM (arrows). AMG/hematoxylin. **(F)** Normal breast tissue, with fine grains of ^AMG^TM (open arrows) attached to the luminal surface of lobule epithelial cells, and particulate ^AMG^TM in scattered epithelial cells (closed arrows). The lumen of one lobule (right) contains black ^AMG^TM-stained secretion (artefactually shrunken); in the left lobule the secretion has fallen out during processing. AMG/hematoxylin (76). **(G)** LA-ICP-MSI of (^AMG^TM-containing) normal breast lobules showing mercury (red/green) in the luminal secretion and epithelium, and iron and nickel (green) in the epithelium (76). **(H)** Breast cancer with numerous neoplastic ductules containing black luminal ^AMG^TM. Enlarged view shows ^AMG^TM in neoplastic duct cells, with ^AMG^TM grains (arrow) attached to the nuclear membrane. ^AMG^TM is present in the ductule lumen (asterisk). AMG/hematoxylin (76).

Autometallography of human kidneys showed ^AMG^TM predominantly in the cortex in renal proximal tubule cells (sparing glomeruli and distal tubule cells) (**Figures 2A, B**), and in the medulla in Henle thin loops (not in collecting ducts) (66, 80). Renal tubule cell ^AMG^TM started appearing in the third decade of life (in 66% of people aged 21–40 years) and peaked at 84% of people aged 61–80 years (77, 80). LA-ICP-MSI indicated that the ^AMG^TM most commonly present was mercury, and demonstrated mixtures of cadmium, lead, nickel, and silver in kidneys (**Figure 2C**) (66). Clear cell carcinoma, the most common type of renal cancer, is thought to arise from the proximal tubules (81), the cells in the kidney that most often harbored toxic metals.

#### 2.2.2 Pancreas

Pancreatic islets, and nearby acinar and ductal cells, readily take up xenobiotics due to their high blood flow and fenestrated capillaries (82). Autometallography showed ^AMG^TM in normal pancreatic islet cells, often in peripheral islet cells and those adjacent to microvessels (**Figure 2D**), in 16% of people without pancreatic cancer and in 53% with pancreatic cancer (65). ^AMG^TM in islets was confined to insulin-producing ß-cells (77), the cell of origin of insulinomas. ^AMG^TM was also seen in acinar cells (**Figure 2D**) in 24% of people with pancreatic cancer, but not in people without pancreatic cancer. Periductal cells containing ^AMG^TM (**Figure 2D**) were present in 11% of people with pancreatic cancer, but not in people without tumors. Islet cells, periductal cells and acinar cells are all potential candidates for pancreatic progenitor cells (83, 84) where mutations could initiate pancreatic cancer. LA-ICP-MSI showed the ^AMG^TM in the pancreas was most often mercury (**Supplementary Figure 1A**). Other metals detected in the pancreas were cadmium, chromium, lead, and nickel (65). ^AMG^TM was seen in some groups of pancreatic cancer cells (**Figure 2E**), and so could promote further mutations leading to tumor clones.

#### 2.2.3 Thyroid

Autometallography showed ^AMG^TM in the cytoplasm of thyroid follicle epithelial cells (**Supplementary Figure 1B**) of 4% of people aged 1–29 years, 9% aged 30–59 years, and 38% aged 60–104 years (85). The density of ^AMG^TM varied both within and between samples. No thyroid cancer was present in any sample. LA-ICP-MSI indicated that mercury was likely to be the cause of most ^AMG^TM positivity (**Supplementary Figure 1C**). Other metals seen in thyroid follicle epithelium were cadmium, lead, and nickel (85). Most thyroid carcinomas are derived from thyroid follicle epithelium (81).

#### 2.2.4 Nervous system

Cells in the human nervous system that contained ^AMG^TM were astrocytes, oligodendrocytes, neurons, pericytes, pinealocytes, choroid plexus cells, and white blood cells (67, 74, 75, 80, 86, 87). *Astrocytes* were the cells most frequently containing ^AMG^TM, often with dense staining of cell bodies and astrocytic processes (**Supplementary Figure 1D**), and involving all four types of astrocytes. Astrocyte-derived tumors (astrocytoma and glioblastoma multiforme) are the most common glial tumors (81). *Oligodendrocytes* often contained ^AMG^TM (**Supplementary Figure 1D**), especially those in grey matter. Oligodendrogliomas are the second most common glial tumor. *Neurons* in the locus ceruleus in the brain stem had a marked age-related tendency to take up and retain toxic metals, with ^AMG^TM starting to be seen in the 20–29 years group (22% of people), and peaking at 67% of people in the 60–69 years group, indicating that toxic metals are commonly taken up by adult brains (71, 88). The locus ceruleus helps maintain the blood-brain-barrier, so toxic metals in this nucleus could impair this barrier and allow other toxicants to pass into the central nervous system to initiate tumors. Other neurons in the brain were less likely than glial cells to take up ^AMG^TM, and neuronal tumors in adults are less common than glial tumors (81). Other nervous system cells that contained ^AMG^TM (and their associated tumors) were *pericytes* (**Supplementary Figure 1D**) (hemangiopericytoma), *pinealocytes* (pineal gland tumor), *choroid plexus cells* (papilloma), and *white blood cells* (primary CNS lymphoma). LA-ICP-MSI indicated that mercury and silver were likely to be the metals responsible for the nervous system ^AMG^TM, though only one person had a known exposure to mercury. LA-ICP-MSI of the locus ceruleus showed neurons harbor different combinations of toxic metals (88), which enables this nucleus to be used to estimate previous exposures to metal toxicants.

#### 2.2.5 Anterior pituitary

Autometallography showed ^AMG^TM in anterior pituitary cells (**Supplementary Figure 1E**) in 33% of people aged 2–20 years, and increased in frequency on aging, reaching a peak of 88% of people in the 61–80 years group (64). Growth-hormone containing somatotrophs were the cells most often containing ^AMG^TM (**Supplementary Figure 1F**). LA-ICP-MSI showed mercury in regions of the pituitary that had ^AMG^TM (**Supplementary Figure 1G**), with no other toxic metals being seen in the pituitary (64). Growth hormone-secreting somatotroph adenomas are a common type of pituitary tumor (81).

#### 2.2.6 Breast

Breast cancer samples removed at surgery showed ^AMG^TM in normal breast tissue apart from the tumor, with ^AMG^TM seen in intraductal secretions and luminal epithelial cells in 55% of samples (76) (**Figure 2F**). LA-ICP-MSI detected mercury in samples that contained ^AMG^TM (**Figure 2G**), and found other metals such as nickel, iron, aluminum, chromium and cadmium in some samples (76). Neoplastic cells containing ^AMG^TM (**Figure 2H**) were seen in 23% of breast cancers. The female breast may be particularly susceptible to cancer because metal transporters ferry toxicants like mercury from the circulation through breast epithelial cells to enter luminal secretions (76). Epithelial progenitor cells undergoing mitoses during an episode of mercury exposure would be at risk for genotoxic damage from mercury, and mercury-rich luminal secretions would expose epithelial cells to mercury long-term (76).

#### 2.3.7 Other organs

In the *ovary* ^AMG^TM was seen attached to nuclei of epithelial cells and in the zona pellucida (**Supplementary Figure 1H**) (77). Fallopian tube tissue [where some ovarian cancers arise (81)] was not available for analysis. Other human organs where ^AMG^TM or LA-ICP-MSI detected toxic metals have been found (and common tumors arising at these sites) are the *liver* (77) hepatocytes (hepatocellular carcinoma) and portal tracts (biliary carcinoma), the *adrenal gland* (78) cortex (adrenal adenoma) and medulla (phaeochromocytoma), and the *retina* (79) (retinoblastoma).

## 3 Discussion

There are two key points of this Perspective. (**1**) Many types of normal human cells contain toxic metals such as mercury, which increase during aging. (**2**) The human cell types containing toxic metals are those susceptible to neoplasia. The finding of toxic metals in human cells can help explain the increasing incidence of some cancers, the increase of cancer incidence with aging, and multiple cancers.

Studies of age-adjusted cancer incidences over time indicate that some cancers are increasing in incidence (89, 90). Furthermore, the incidence of colorectal, breast, kidney, pancreas, and uterine cancer is increasing in younger age groups (91, 92). One possible reason for increases in cancer incidence could be increasing atmospheric, water and soil pollution with carcinogenic toxic elements (2). For example, increased atmospheric pollution with mercury from burning fossil fuels and artisanal gold mining (20, 93) leads to increased human mercury intake, both with mercury vapor inhalation and with methylmercury ingestion of mercury-contaminated fish (94), with global fish consumption now outstripping human population growth (95).

The incidence of most adult cancers increases with increasing age (81), so it is of interest that the prevalence of people with ^AMG^TM in cells of the kidney, thyroid, anterior pituitary, pancreas, adrenal medulla, and brain neurons also increases during aging (77). Another potential reason for the later age of onset of most adult cancers is that cellular methylmercury is slowly demethylated in the body to more toxic inorganic mercury (96), so the genotoxic potential of intracellular mercury increases with increasing age. A puzzling phenomenon is the *decrease* in mortality for many cancers in advanced older age (97, 98), which could contribute to the plateau of mortality in advanced age (99, 100). One reason for this could be that the proportion of people having detectable ^AMG^TM in cancer-prone cells falls over the age of 80 years (77), so people who have been exposed to less metal toxicant during their lives could be less likely to develop cancer later in life.

People with ^AMG^TM in one organ usually have toxic metals in several other organs as well (77). This raises the possibility that exposure to metal toxicants could contribute to the occurrence of concurrent multiple primary tumors, in conjunction with genetic susceptibilities to these cancers (101).

Exposing experimental animals to toxic metals (usually mercury) and then biomapping the metal location with autometallography has given valuable insights into the types of cells taking up toxic metals. (**1**) Autometallography has shown ^AMG^TM within the same cell types in animals as contain ^AMG^TM in humans (**Supplementary Table 2**). For example, in animals exposed to mercury, silver or bismuth, ^AMG^TM was seen in the same pattern as human cells in the kidney (63, 102–109), pancreas (62, 102, 104, 109), thyroid (103, 104, 110), nervous system (37, 63, 102–104, 109, 111–114), pituitary (102, 104, 109, 110, 115, 116), ovary (62, 104), liver (47, 102–106, 117), adrenal gland (58, 62, 102, 104), retina (111), white cells (47, 62, 102, 104), and endothelial cells (47, 58, 103, 104, 112, 118, 119). Even exposure to low levels of mercury from a few dental amalgam fillings in non-human primates resulted in the widespread cellular uptake of mercury (102). (**2**) Autometallography combined with electron microscopy showed ^AMG^TM from mercury or silver exposure in lysosomes, mitochondria, Golgi apparatus, endothelial basement membrane, and nuclear membrane, as well as within nuclear euchromatin and nucleoli (37, 47, 58, 62, 63, 87, 102, 104, 112, 114, 116, 120–128), all subcellular sites implicated in neoplasia (129) (**Supplementary Table 2**). (**3**) Mercury vapor and methylmercury pass readily through the placenta and enter the developing fetus (52, 55). After gestational exposure to mercury vapor, ^AMG^TM was found in neonatal mouse renal tubule cells, liver periportal cells, synovial cells, chondrocytes, retinal cells, optic nerve glial cells, and fibroblast progenitor cells (111, 117), indicating toxic metals can be taken up preferentially by developing cells (**Supplementary Table 2**). These findings suggest that prenatal toxic metal exposure could plant the seed for later carcinogenesis, either via mutations or epigenetic changes in developing cells, and could contribute to the pathogenesis of early-onset cancers such as retinoblastoma, optic nerve glioma, and soft tissue sarcomas (81).

Some human autopsy tissue is not available for elemental analysis because of limited routine organ sampling. Here small animal autoradiography using radiolabeled isotopes is useful, since it has shown the widespread organ uptake (including the colon, esophagus, lacrimal and salivary glands, bone marrow, fat, and muscle) of different forms of mercury, and the length of time mercury persists within organs (51–55) (**Supplementary Table 2**). Autoradiography can map the routes inhaled mercury vapor takes through the body (via the lungs, blood, kidneys, liver, bile duct, and gastrointestinal tract), and that ingested methylmercury takes (via the gastrointestinal tract, blood, liver, bile duct, and colon) (56). Mercury therefore passes through many of the organs where cancers frequently arise (**Supplementary Figure 2**).

Future projects on the role of toxic metals in cancer could be: (**1**) Since toxic metals can be transported across the placenta into the fetus (130), animal studies could be used to find if ^AMG^TM is present in a wide range of fetal stem or progenitor cells (131). (**2**) Elemental biomapping of biopsied tissues not routinely sampled at autopsy, such as the fallopian tubes, could be undertaken. (**3**) DNA damage has been reported for metals other than mercury, including cadmium (132, 133) and silver (134). Toxicity synergy exists for several metals (3, 135) so exposure to multiple toxic metals may be more potent in promoting carcinogenesis than single metals alone (4). Future experimental studies therefore need to examine the carcinogenic effect of multiple metal toxicants. (**4**) Toxicologists could take a leaf out of the genetics playbook (where whole genome or exome sequencing has largely replaced searches for individual gene variants) by greatly expanding the range of potentially toxic elements to be biomapped in tissues. (**5**) Potentially-toxic metals acting alone are unlikely to be the sole cause of most cancers, since in humans many organs contain ^AMG^TM in later adult life (77), whereas cancers arise in only a proportion of these. It is likely that genetic susceptibilities to environmental toxicants are present in a majority of cancers, so combined next-generation genetic analyses and extensive toxic element biomapping will be needed to uncover these interactions (1).

In conclusion, human elemental biomapping shows that potentially-carcinogenic toxic metals are present in many of cells from which common tumors arise. The increasing global incidence of many tumors could be associated with increasing toxic metal pollution from anthropogenic toxic metal pollution of the atmosphere, water and soil. More work is needed to confidently assign the roles of toxic metals to common cancers, in particular looking for gene-toxic metal interactions. However, a precautionary approach to reduce the incidence of cancers would be to reduce toxic metal-emitting industrial and mining activities and the burning of fossil fuels.

## Data availability statement

The original contributions presented in the study are included in the article/**Supplementary Material**. Further inquiries can be directed to the corresponding author.

## Ethics statement

The studies involving humans were approved by the Human Ethics Committee, Sydney Local Health District (Royal Prince Alfred Branch). The studies were conducted in accordance with the local legislation and institutional requirements. The human samples used in this study were acquired from previous studies for which ethical approval was obtained. Written informed consent for participation was not required from the participants or the participants’ legal guardians/next of kin in accordance with the national legislation and institutional requirements.

## Author contributions

RP: Conceptualization, Data curation, Formal analysis, Funding acquisition, Investigation, Methodology, Project administration, Resources, Visualization, Writing – original draft, Writing – review & editing. DPB: Data curation, Formal analysis, Investigation, Methodology, Resources, Visualization, Writing – review & editing.

## Supplementary material

Supplementary Figure 1 Toxic metals in the human pancreas, thyroid, brain, pituitary, and ovary. **(A)** LA-ICP-MSI shows the location of pancreatic islets demonstrated by zinc imaging (left panel). Mercury (red/green) is seen in the same islets (right panel) that contained ^AMG^TM (65). **(B)** A normal thyroid with dense ^AMG^TM in most thyroid follicle epithelial cells. *Inset*: magnified view showing ^AMG^TM (arrows) within the cytoplasm of thyroid follicle epithelial cells. AMG/hematoxylin (85). **(C)** LA-ICP-MSI shows mercury (red/green) in thyroid follicle epithelium that contained ^AMG^TM (85). **(D)** Cerebral white matter where the cell bodies of brown-stained fibrous astrocytes contain dense black ^AMG^TM (open arrowheads). ^AMG^TM grains are seen in astrocyte processes (closed arrowheads) which end on blood vessels (BV). *Right inset*: a cortical oligodendrocyte contains perinuclear ^AMG^TM (arrowhead). *Left inset*: ^AMG^TM in brain pericytes (closed arrowheads) and an endothelial cell (open arrowhead). AMG/glial fibrillary acidic protein (AMG/Luxol fast blue in left inset) (67, 75). **(E)** Anterior pituitary cells containing ^AMG^TM granules. AMG/hematoxylin (64). **(F)** Anterior pituitary growth hormone-stained (red) somatotrophs contain ^AMG^TM granules (closed arrows), but not cells without growth hormone (open arrows). AMG/growth hormone/hematoxylin (64). **(G)** LA-ICP-MSI shows mercury (red/yellow) in a region of the anterior pituitary with ^AMG^TM-containing cells (right lower panel), but not in an ^AMG^TM-free region (right upper panel) (64). Scale = counts per second (proportional to abundance). **(H)** Normal ovarian follicle with ^AMG^TM grains attached to nuclei of epithelial cells (arrowhead in enlargement), in the zona pellucida (arrow), and in round profiles (asterisk) in the antrum. AMG/hematoxylin (77).

Supplementary Figure 2 Cellular pathways in the body of mercury vapor (Hg^0^) and methylmercury (MeHg+). **(A)** Inhaled Hg^0^ is taken up by the lungs, and absorbed by plasma, red blood cells, and cells in multiple organs, where conversion to toxic inorganic mercury (Hg^2+^) takes place. Excretion of Hg^2+^ is via the kidney and urine, and via the liver, bile duct, and gastrointestinal tract (GIT) and excreted through the feces. **(B)** Ingestion of dietary MeHg+ by the GIT leads to MeHg+ being taken up by the blood and then either (i) passes into cells of multiple organs where it is slowly converted to Hg^2+^, or (ii) is re-circulated into the GIT by the hepato-biliary pathway. MeHg+ in the colon is rapidly converted to Hg^2+^ by colonic flora and excreted in the feces. In both pathways toxic Hg^2+^ is deposited or passes through many of the cells that are prone to cancer.
