# Supplementary material for: Elemental biomapping of human tissues suggests toxic metals such as mercury play a role in the pathogenesis of cancer
Source: Front Oncol. 2024 Jun 21;14:1420451. doi: 10.3389/fonc.2024.1420451 (PMC11224479; doi:10.3389/fonc.2024.1420451)
Supplement: Supplementary file 3 [file Table_1.docx]

**Supplementary Table 1**. Carcinogenic mechanisms of action of lead, cadmium, mercury, arsenic, and chromium. References are listed in the article.

| **Carcinogenic mechanism** | **Pb** | **Cd** | **Hg** | **As** | **Cr** |
| --- | --- | --- | --- | --- | --- |
| DNA damage | (3,5,18,19) | (3,18,19) | (3,5,13,18,19) | (3,5) | (3,5) |
| DNA impaired repair | (3,18,19) | (3,5,18) | (3,5,13,18) | (3,5) | (5) |
| Inflammation and oxidative stress | (3,5,18,19) | (3,5,18,19) | (3,5,13,18) | (3,5,19) | (3,5) |
| Epigenetic changes | (5,19) | (18,19) |  | (3,5) |  |
| Increased cell survival and proliferation | (18,19) | (3,5,18) | (5,19) | (3,5,19) | (5) |
| Damage to organelles and membranes | (5,19) | (3,18) | (5,13,18,19) | (3,5) |  |
