# Supplementary material for: Elemental biomapping of human tissues suggests toxic metals such as mercury play a role in the pathogenesis of cancer
Source: Front Oncol. 2024 Jun 21;14:1420451. doi: 10.3389/fonc.2024.1420451 (PMC11224479; doi:10.3389/fonc.2024.1420451)
Supplement: Supplementary file 4 [file Table_2.docx]

**Supplementary Table 2.** Organ and cell origin of neoplasms, and references to the locations of biomapped toxic metals in the tissues of humans and experimental animals.

| **Organ or cell origin of neoplasms** | **Neoplasms*** | **Toxic metal located in organs or cells** |
| --- | --- | --- |
| Adrenal cortex | Adenoma | Cortical cells (51,52,58,78,104) |
| Adrenal medulla | Pheochromocytoma | Chromaffin cells (54,62,78,102,104,128) |
| Anterior pituitary | Adenoma | Growth hormone-containing cells (54,64,102,104,116) |
| Biliary tract epithelium | Carcinoma | Epithelium (62) |
| Blood vessel endothelium | Sarcoma | Endothelium# (47,54,58,62,75,104,111,112,114,117-119,121,136) |
| Breast epithelium | Carcinoma | Epithelium (55,76) |
| Chondrocyte | Sarcoma | Chondrocytes (117) |
| CNS astrocyte | Astrocytoma/GBM | Astrocytes (53,54,67,75,79,87,103,104,111-114,120-124,136-139) |
| CNS choroid plexus | Papilloma | Choroid plexus cells (55,112,114,119,120,123,124,136,140) |
| CNS ependyma | Ependymoma | Ependymal cells (104,119,122-124,136,137,140) |
| CNS neuron | Neuroblastoma | Neurons (37,58,62,63,67,74,75,104,112-114,119,120,122-124,136,137,139,140) |
| CNS oligodendrocyte | Oligodendroglioma | Oligodendrocytes (37,67,74,75,112) |
| Colon | Carcinoma | Colon (51-55,103,128,138) |
| Esophagus | Carcinoma | Esophagus (55) |
| Fat cell | Sarcoma | Fat (51,141) |
| Fibroblast | Sarcoma | Fibroblasts (62,104,117) |
| Gall bladder | Carcinoma | Gall bladder (55,62) |
| Heart | Myxoma | Myocardium (51,52,55,138) |
| Kidney epithelium | Carcinoma | Epithelium (51-55,58,62,63,66,102-108,117,138,142) |
| Lacrimal gland | Carcinoma | Lacrimal gland (53,54) |
| Liver hepatocyte | Carcinoma | Hepatocytes (47,51-53,55,58,62,102-106,117,138) |
| Liver Kupffer cell | Sarcoma | Kupffer cells (62) |
| Lung alveolar cell | Carcinoma | Alveolar cells (51,52,55,102,108,112,138,143) |
| Lung epithelium | Carcinoma | Bronchus epithelium (52) |
| Lymph node/white cell | Lymphoma/leukemia | Lymph node/white cells (47,52,54,55,58,62,102,104) |
| Mast cell | Mastocytosis | Mast cells (58,104) |
| Meninges arachnoid | Meningioma | Arachnoid cells (103,120) |
| Muscle striated | Sarcoma | Muscle striated (52-54,86,104,106) |
| Nasal mucosa | Carcinoma | Nasal mucosa (51,52,55) |
| Nerve perineurium | Perineurioma | Perineurium (128) |
| Nerve Schwann cell | Schwannoma | Schwann cells (62,125,128) |
| Ovary epithelium | Carcinoma | Epithelium (51,62,77,104) |
| Pancreas epithelium | Carcinoma | Epithelium (52,55,65) |
| Pancreas β cell | Insulinoma | β cells (52,65,104) |
| Pericyte | Hemangiopericytoma | Pericytes (75,114) |
| Pineal pinealocyte | Pineoblastoma | Pinealocytes (114) |
| Placenta | Chorioangioma | Placenta (54,55) |
| Prostate | Carcinoma | Prostate (55) |
| Retina | Retinoblastoma | Retinal cells (51,79,111,113,121,144) |
| Salivary gland | Adenoma | Salivary gland (52-55) |
| Skin | Various | Skin (54,55) |
| Spleen white blood cell | Lymphoma | Spleen white blood cell (52-55) |
| Stomach | Carcinoma | Stomach (53-55) |
| Synovium cell | Sarcoma | Synovial cells (117) |
| Testis Leydig cell | Leydig cell tumor | Leydig cells (126,145,146) |
| Testis Sertoli cell | Sertoli cell tumor | Sertoli cells (126,127,145) |
| Testis sperm line | Seminoma | Sperm cells (55,58,127,138) |
| Thymus | Thymoma | Thymus (54,55) |
| Thyroid C cell | Carcinoma | C cells (104) |
| Thyroid epithelium | Carcinoma | Epithelium (52,85,103,104) |
| Trachea | Carcinoma | Trachea (51) |
| Urinary bladder epithelium | Carcinoma | Epithelium (54,103,104) |
| Uterus | Carcinoma | Uterus (54,55) |
| Vagina | Carcinoma | Vagina (54,55) |

* Benign neoplasm listed where malignant neoplasm is rare (81). GBM glioblastoma multiforme, # seen in most AMG-positive samples. **Species**: *Human* (64-67,74-79,85-87,118,146). *Non-human primate* (52,102,113,139,144). *Rat or mouse* (37,47,51-55,58,62,63,107,108,112,114,116,117,119-121,123-128,136-138,140-142,145). *Dog* (103,104,128). *Whale* (105,106). *Guinea pig* (143). **Techniques**: *Autoradiography* (51-55,107,138,140-143). *AMG* (37,47,58,62-64,66,67,74-79,86,87,102-106,112,114,116-123,125-128,136-139,144,146). *AMG & electron microscopy* (37,47,58,62,63,87,102,104,112,114,116,120-128). *LA-ICP-MSI* (64-66,74-76,78,79,86,118). *Silver exposure* (58,62,114,120,121,127,128). *Bismuth exposure* (63,87,108,145) (all other exposures to mercury). *Mercury gestational exposure* (111,113,117,120,141).

**References**

References 1-135 are listed in the article.

136. Moller-Madsen B, Danscher G. Localization of mercury in CNS of the rat. IV. The effect of selenium on orally administered organic and inorganic mercury. *Toxicol Appl Pharmacol* (1991); 108:457-73. doi: 10.1016/0041-008x(91)90092-s

137. Moller-Madsen B, Danscher G. Localization of mercury in CNS of the rat. I. Mercuric chloride (HgCl2) per os. *Environ Res* (1986); 41:29-43. doi: 10.1016/s0013-9351(86)80165-7

138. Berlin M, Johansson LG. Mercury in mouse brain after inhalation of mercury vapour and after intravenous injection of mercury salt. *Nature* (1964); 204:85-6.

139. Warfvinge K, Hua J, Logdberg B. Mercury distribution in cortical areas and fiber systems of the neonatal and maternal adult cerebrum after exposure of pregnant squirrel monkeys to mercury vapor. *Environ Res* (1994); 67:196-208. doi: 10.1006/enrs.1994.1074

140. Cassano GB, Viola PL, Ghetti B, Amaducci L. The distribution of inhaled mercury(Hg203) vapors in the brain of rats and mice. *J Neuropathol Exp Neurol* (1969); 28:308-20.

141. Danielsson BR, Khayat A, Dencker L. Foetal and maternal distribution of inhaled mercury vapour in pregnant mice: influence of selenite and dithiocarbamates. *Pharmacol Toxicol* (1990); 67:222-6. doi: 10.1111/j.1600-0773.1990.tb00817.x

142. Rodier PM, Kates B. Histological localization of methylmercury in mouse brain and kidney by emulsion autoradiography of 203Hg. *Toxicol Appl Pharmacol* (1988); 92:224-34.

143. Berlin MH, Nordberg GF, Serenius F. On the site and mechanism of mercury vapor resorption in the lung. A study in the guinea pig using mercuric nitrate Hg 203. *Arch Environ Health* (1969); 18:42-50. doi: 10.1080/00039896.1969.10665370

144. Warfvinge K, Bruun A. Mercury accumulation in the squirrel monkey eye after mercury vapour exposure. *Toxicology* (1996); 107:189-200. doi: 10.1016/0300-483x(95)03257-g

145. Stoltenberg M, Danscher G, Pamphlett R, Christensen MM, Rungby J. Histochemical tracing of bismuth in testis from rats exposed intraperitoneally to bismuth subnitrate. *Reprod Toxicol* (2000); 14:65-71. doi: 10.1016/s0890-6238(99)00060-x

146. Keck C, Bergmann M, Ernst E, Muller C, Kliesch S, Nieschlag E. Autometallographic detection of mercury in testicular tissue of an infertile man exposed to mercury vapor. *Reprod Toxicol* (1993); 7:469-75. doi: 10.1016/0890-6238(93)90092-l
